# Nutritive Value of the Different Parts of the Grain of Wheat

**Published:** 1885-11

**Authors:** 


### Nutritive Value of the Different Parts of the Grain of Wheat.

M. Aime Girard has recently contributed an interesting article on this subject to the *Annales de Chimie et de Physique*. He treats the grain of wheat as consisting essentially of three parts: (1) The integument, including not only the pericarp, but also the outer envelopes of the endosperm or albumen; (2) the embryo, separated from the endosperm; and (3) the mealy endosperm, freed from its outer envelopes. He discusses these three constituents in detail, from the points of view of their anatomical and chemical composition, the part taken by each in the composition of bread, and their properties in relation to digestion; the general result being that the endosperm is the only part of the grain which is of value for nutritive purposes without a compensating drawback, the integuments and embryo being either useless or actually injurious.

The integument, which makes up 14.36 per cent. of the entire grain, is rich in nitrogenous substances, to the extent of 18.75 per cent., but these substances are only to a very small extent soluble in the digestive apparatus of man; the portion that is thus assimilable being only 0·4 per cent of the whole grain. Among these nitrogenous substances is the cerealin discovered by Mege-Mouries, the ferment which causes the formation of black bread. In addition, there are mineral substances which are soluble in the gastric juice to the extent of 0.45 per cent. of the grain.

The embryo contains a still larger proportion of nitrogenous substance than the integument, and especially of such as are apparently assimilable. But it also is of scarcely any service for nutrition, because of the large proportion which it contains of the injurious cerealin, in addition to a very easily oxidizable oil, which easily escapes from the cells in which it is formed, distributes itself through the flour, and assists in its decomposition. The maximum amount in the embryo and integument together serviceable for nutrition is 1.0 per cent. of nitrogenous and 0.5 per cent. of mineral substances. And

